# Editorial: Neural implementation of expertise

**DOI:** 10.3389/fnhum.2015.00545

**Published:** 2015-09-30

**Authors:** Merim Bilalić, Robert Langner, Guillermo Campitelli, Luca Turella, Wolfgang Grodd

**Affiliations:** ^1^Department of Cognitive Psychology, Alps Adria University KlagenfurtKlagenfurt, Austria; ^2^Clinical Neuroscience and Medical Psychology, Heinrich Heine University DüsseldorfDüsseldorf, Germany; ^3^School of Psychology and Social Science, Edith Cowan UniversityPerth, WA, Australia; ^4^Center for Mind/Brain Sciences, University of TrentoTrento, Italy; ^5^Department of Magnetic Resonance, Max Planck Institute for Biological CyberneticsTuebingen, Germany

**Keywords:** expertise, neurosciences, sports, music, chess, go, language, perception

How the brain enables humans to reach an outstanding level of performance typical of expertise is of great interest to cognitive neuroscience, as demonstrated by the number and diversity of the articles in this Research Topic (RT). The RT presents a collection of 23 articles written by 80 authors on traditional expertise topics such as sport, board games, and music, but also on the expertise aspects of everyday skills, such as language and the perception of faces and objects. Just as the topics in the RT are diverse, so are the neuroimaging techniques employed and the article formats. Here we will briefly summarize the articles published in the RT.

## Board games

The traditional expertise domain of board games has been covered in the RT by two articles, both employing the expertise approach of pitting experts against novices (Bilalić et al., 2010, 2012, 2014) but employing differing neuroimaging techniques. Bartlett et al. (2013) employed fMRI to demonstrate that chess experts engage the fronto-parietal network when they try to find a logical pattern in a “constellation” of randomly placed chess pieces. Jung et al. (2013) found structural differences as well as differences in brain networks between Baduk (Korean name for the board game Go) experts and novices, which point out the importance of visuospatial processing in problem solving and decision making of board-game experts.

## Sport

Wright et al. (2013) extended the research on anticipation of action in sport by showing that the neural basis for deception involves, besides the well-known action observation network, the structures responsible for social cognition and affection. Turella et al. (2013) review other recent studies on the anticipation of action in sport and connect them with the mirror neurons in animal research. The review by Chang (2014) deals with motor domains such as sports and music and the structural and functional changes associated with expertise. Debarnot et al. (2014) go a step further in their review and contrast the neural changes during skill acquisition with those in mental training techniques such motor imagery and mediation.

## Music

Music has been one of the most often investigated domains in expertise because its complexity and richness enable researchers to tackle diverse topics. The variety of themes in the domain of music is also evident in this RT. Tervaniemi et al. (2014), for example, pitted expert musicians against novices in a novel paradigm to investigate memory and attentional processes with EEG. On the other hand, Bergman Nutley et al. (2014) used the music domain to investigate longitudinal effects on cognitive processes such as working memory, speed of processing, and reasoning, while Fauvel et al. (2013) apply the promising findings of transfer and neural plasticity associated with musical practice to cognitive aging in their review.

## Language

Unlike the previous articles, which deal with specialized expertise domains, a number of contributions highlight the fact that even the everyday skills we often take for granted represent impressive feats of human expertise. One group of articles deals with language, which is one such everyday skill. Reichle and Reingold (2013) review the electrophysiological evidence of the link between eye movements and the mind during reading. The learning of a second language based on its similarity to one's native language was investigated by Grimaldi et al. (2014), while Dietrich et al. (2013) demonstrated the neural changes associated with the process of learning to comprehend speech that was several times faster than normal speech. Finally, Lotze et al. (2014) demonstrate by means of resting-state fMRI that people who write highly creatively have increased functional connectivity between the task-related brain regions in the right hemisphere but reduced interhemispheric connectivity.

## Perception

Similarly, a couple of articles deal with perception of own-race and other-race faces (Wiese, 2013) as well as with perception of familiar faces and objects and the functional connectivity within the medial temporal lobe (McLelland et al., 2014). The role of the fusiform face area (FFA) in expertise has been a bone of contention between Harel et al. (2013, 2014), on the one hand, and Wong and Wong (2014), on the other.

## Theoretical and simulation work

Finally, a number of articles provide either new theoretical ideas or revisions of already established theories. Campitelli and Speelman (2013) highlight the advantages of using the expertise paradigm in investigating memory, while Brogliato et al. (2014) expand the Sparse Distributed Memory (SDM) model to incorporate the effects of practice on memory retrieval. Guida et al. (2013) extend their two-stage framework of skill acquisition (Guida et al., 2012) by arguing for the functional cerebral reorganization (FCR) as being the neural signature of expertise. The way one structures training studies is considered by Coffey and Herholz (2013), who suggest a new approach for characterizing and deconstructing the task requirements in training studies. Finally, Harré (2013) demonstrates the parallels between two seemingly unrelated fields, perceptual expertise and social cognition.

## Conclusion

It is clear that we cannot do justice to all submissions in this brief editorial. We hope, however, that our brief summary demonstrates the diversity in topics and methods employed in research on human expertise and also, indirectly, the growing interest in the field of expertise. It should become evident that research on expertise is not only relevant for understanding exceptional human performance but also for understanding how mind and brain work more generally. We are grateful to all authors for their contribution and hope that the RT, with its broad and deep coverage, will provide a useful reference for the reader interested in expertise and, particularly, current approaches to its neural implementation.

### Conflict of interest statement

The authors declare that the research was conducted in the absence of any commercial or financial relationships that could be construed as a potential conflict of interest.
